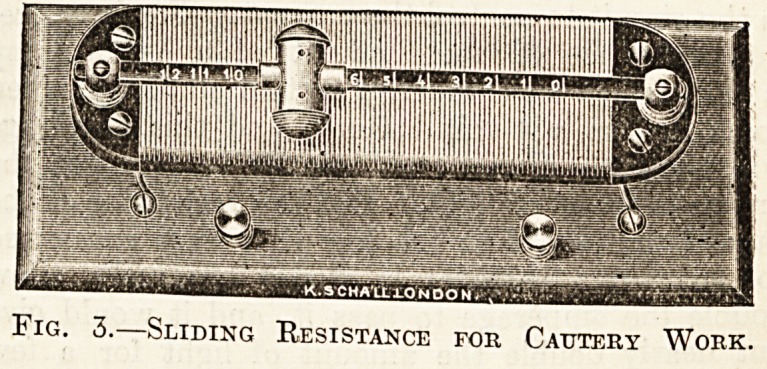# Apparatus for Cautery and Light

**Published:** 1911-12-30

**Authors:** Alfred C. Norman

**Affiliations:** House Surgeon at Sunderland and Durham County Eye Infirmary; late House Surgeon in Charge of *x*-ray Department, Royal Buckinghamshire Hospital.


### ELECTRICITY IN MODERN MEDICINE.

### IV.—Apparatus for Cautery and Light.

By ALFRED C. NORMAN, M.D., Edin., House Surgeon at Sunderland and Durham County Eye Infirmary; late House Surgeon in Charge of *x*-ray Department, Royal Buckinghamshire Hospital.

THE *galvano cautery* depends upon the fact that an electric current produces heat in overcoming the resistance of a circuit. (Joule's law tells us that the larger the current and the higher the resistance the greater will be the heat produced.) The tissues are burnt by the hot metal and not by the electricity: the voltage employed is too low and the alternative path back through the cautery-burner too easy for any appreciable current to enter the body.

### SIZE OF THE BURNER.

The very large burners used for gynæcological purposes require as much as fifty ampères to bring them to a red heat; they are most conveniently worked by a transformed current from the main. The tiny burners employed by ophthalmic surgeons consume no more than seven or eight ampères, to supply which a single bichromate bottle-cell of two-quarts size would suffice, though an accumulator is more satisfactory. We shall consider here only cauteries of medium size (requiring a current of 12 to 18 ampères) suitable for ear, nose, and throat work and for most ordinary purposes, such as the destruction of nævi. The following apparatus is necessary: a battery (or else a transformer), a pair of connecting cords, a handle, some burners, and a variable resistance.

*The battery.*—Suitable accumulators and bichromate batteries have been described on pages

FIG. 1.—CAUTERY HANDLE FOR GENERAL PURPOSES.

252 and 202 respectively. Transformers will be discussed in a later section.

*The connecting cords* must be stout or they will be heated by the large ampèrage they have to carry. To be as flexible as possible they should consist of many fine wires twisted into a strand and insulated by a covering of woven cotton.

*The cautery-handle* may be a "universal" one to take snares as well as ordinary burners, or a "simple" one for burners alone; the latter is sufficient for most purposes. There should be a spring contact-maker, operated by a button or trigger, so that the current may be cut off immediately the operation is finished. Fig. 1 illustrates Dr. Schech's pattern of simple handle. The writer's simple handle for eye work is seen in front of the accumulator on page 251.

*The burner* consists of a loop of platinum wire which has been flattened out to any required shape and its ends fused to two pieces of thick copper wire, insulated from each other by turns of silk thread (fig. 2). In the handle are two insulated metal rods, the distal ends of which must be connected to the battery by means of the cords; the burner fits into their proximal ends, and so completes the circuit. To be serviceable for surgical work the platinum loop has to be fairly stout, hence its resistance is actually very low, but it is relatively high for the large current that passes:

FIG. 2.—CAUTERY BURNERS.

consequently it is the only part of the circuit that becomes hot.

A cautery-handle, three assorted burners, and a pair of cords in leather case cost about 29s.

### SLIDING RESISTANCE.

*The variable resistance.*—Extra resistance must be put in the circuit to control the current, because, even with a 2-volt accumulator, there would be far too great an ampèrage through the burner if the cautery were connected directly to the battery. Taking the total resistance of cords, handle, and burner as .06 ohm and the internal resistance of the accumulator as .003 ohm, the current would be 31 ampères,

$$\left(\frac{2\text{ volts}}{\cdot06\times\cdot003\text{ ohm}}\right)=31\text{ ampères,}$$

which would bring to a white heat, or even melt, the burner. The resistance may be put in at any part of the circuit, but it is conveniently connected between the end of one of the cords and either terminal of the battery. The wire of which it is made must be thick, to carry the current without becoming too hot, but a fraction of an ohm will

FIG. 3.—SLIDING RESISTANCE FOR CAUTERY WORK.

serve since the voltage employed is so low. A suitable resistance may be improvised for a 2-volt accumulator by fixing to an end of one of the cords a piece of the so-called "platinoid" wire (size No. 15, 8 inches long), which is composed of a new alloy having a very high resistance. The wire can then be used as a variable resistance by sliding it through the hole in the battery terminal until a point is reached at which the burner is heated to the desired degree of redness.

The writer's own pattern of resistance with sliding contact is shown connected to the battery on page 251; it costs about 6s. 6d. For batteries of higher voltage we must have a more substantial resistance, such as fig. 3, costing about £1.

All the above are *series* resistances; the principle of *shunt* resistance will be discussed later.

### The Heat of a Cautery.

A cautery should never be used at more than a dull-red heat, or it will sever the tissues like a knife and cause bleeding. It is best to start with all the resistance in circuit, and gradually to move the sliding contact until the desired heat is obtained in the burner.

The large current is a strain on the battery, and must not be left running a second longer than is necessary.

If the burner fail to heat up it may be that its wires have been accidentally bent so that they touch at some point, causing a short circuit; they can be separated with a penknife.

The cords should always be connected first to the cautery-handle before they are joined to the battery; this avoids short-circuiting should their free ends happen to touch in the process of joining them up to the handle.

### Instruments for Electric Light.

The incandescent electric lamp consists of a thin filament of carbon, or of one of the rarer metals such as tungsten, enclosed in a glass bulb which has been exhausted to form a partial vacuum. Under these conditions the filament gives out a bright light when its temperature is sufficiently raised by passing a current of electricity through it.

Electric lamps consume considerably less current than cauteries, but more than we ever require to use in electro-therapeutics; the small lamps used in medical instruments take from ¼ to 1 ampère to bring them to their normal condition of incandescence.

Every lamp is specially made for direct use on a certain voltage, and it must not be used on any other voltage without a suitable resistance in the circuit to control the current. For instance, the resistance of the filament of a 4-volt lamp is designed to let through a certain ampèrage when connected to a 4-volt battery, and this ampèrage will heat the filament sufficiently to give out the proper amount of light. But the resistance of the same lamp would be too low if it were connected to an 8-volt battery; it would obviously allow double the ampèrage to pass it, and it would give out nearly double the amount of light for a few seconds, but the great heat would quickly destroy the filament. If a 4-volt lamp were connected to the 220-volt house-mains the rush of current would be so great that the lamp would explode. On the other hand, a 2-volt battery could not force sufficient ampèrage through a 4-volt lamp to give more than a dull-red glow in the filament.

### How to Vary Candle-power.

By judiciously selecting the thickness and length of the filaments the makers can produce lamps of various candle-power for connecting directly to a supply of any voltage from 2 to 250. The higher the voltage the greater must be the resistance of the filament in order to control and limit the ampèrage passing; consequently high-voltage lamps have long thin filaments. For medical purposes we must have small lamps, and there is not room inside these tiny bulbs for long filaments, therefore we cannot use them directly on the high-voltage house-mains. The house current can however be adapted to the smallest lamps used in medicine by means of a shunt resistance or by transformers; these methods will be discussed in a later section.

If we decide to use a battery as our source of electricity for small lamps, we may choose between accumulators, Leclanché cells, and bichromate cells. The most satisfactory method is to order 4-volt metallic-filament lamps with all our instruments, and to work them from a 4-volt battery—we can thus dispense with a variable resistance, though the small one shown in fig. 4 is inexpensive and very useful for cutting down the light for certain purposes.

*Accumulators* are most satisfactory and cheapest in the long run.

## Figures and Tables

**Fig. 1. f1:**
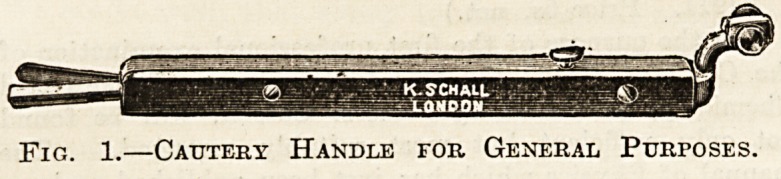


**Fig. 2. f2:**
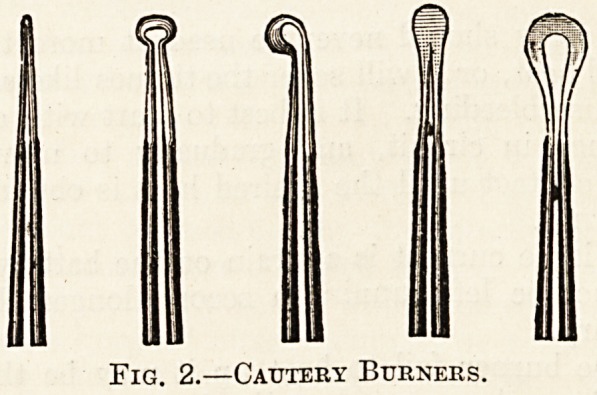


**Fig. 3. f3:**